# Aging steepens the slope of power spectrum density of 30-minute continuous blood pressure recording in healthy human subjects

**DOI:** 10.1371/journal.pone.0248428

**Published:** 2021-03-18

**Authors:** Jumpei Mano, Keita Saku, Hiroyuki Kinoshita, Hiroshi Mannoji, Shigehiko Kanaya, Kenji Sunagawa

**Affiliations:** 1 Graduate School of Science and Technology, Nara Institute of Science and Technology, Nara, Japan; 2 Technology Development HQ, OMRON Healthcare Co., Ltd., Kyoto, Japan; 3 Department of Cardiovascular Dynamics, National Cerebral and Cardiovascular Center Research Institute, Osaka, Japan; 4 Department of Cardiovascular Medicine, Graduate School of Medical Sciences, Kyushu University, Fukuoka, Japan; 5 Circulatory System Research Foundation, Fukuoka, Japan; 6 Department of Therapeutic Regulation of Cardiovascular Homeostasis, Center for Disruptive Cardiovascular Medicine, Kyushu University, Fukuoka, Japan; University of Perugia, ITALY

## Abstract

**Background:**

The increase of blood pressure (BP) variability (BPV) is recognized as an important additional cardiovascular risk factor in both normotensive subjects and hypertensive patients. Aging-induced atherosclerosis and autonomic dysfunction impair the baroreflex and, in turn, augment 24-hour BPV. In small and large animal experiments, impaired baroreflex steepens the slope of the power spectrum density (PSD) of continuous BP in the frequency range of 0.01 to 0.1 Hz. Although the repeated oscillometric BP recording over 24 hours or longer is a prerequisite to quantify BPV in humans, how the very short-term continuous BP recording reflects BPV remains unknown. This study aimed to evaluate the impact of aging on the very short-term (30-min) BPV in healthy human subjects by frequency analysis.

**Methods:**

We recorded continuous BP tonometrically for 30 min in 56 healthy subjects aged between 28 and 85 years. Considering the frequency-dependence of the baroreflex dynamic function, we estimated the PSD of BP in the frequency range of 0.01 to 0.1 Hz, and compared the characteristics of PSD among four age groups (26–40, 41–55, 56–70 and 71–85 years).

**Results:**

Aging did not significantly alter mean and standard deviation (SD) of BP among four age groups. PSD was nearly flat around 0.01 Hz and decreased gradually as the frequency increased. The slope of PSD between 0.01 and 0.1 Hz was steeper in older subjects (71 years or older) than in younger subjects (55 years or younger) (p < 0.05).

**Conclusions:**

Aging steepened the slope of PSD of BP between 0.01 and 0.1 Hz. This phenomenon may partly be related to the deterioration of the baroreflex in older subjects. Our proposed method to evaluate very short-term continuous BP recordings may contribute to the stratification of BPV.

## Introduction

Cardiovascular disease accounts for more than 17 million deaths per year worldwide [1], and appropriate management of blood pressure (BP) is essential to prevent serious complications. The absolute value of oscillometric BP measurement has been used for risk stratification. Recently, BP variability (BPV) has been recognized as an independent predictor of cardiovascular mortality in both normotensive subjects and hypertensive patients [2–9]. Kawai et al. [3] reported that the visit-to-visit BPV using office BP measurement correlated significantly with the incidence of cardiovascular disease. Kikuya et al. [7] also reported that daily BPV assessed by standard deviation (SD) of 24-h ambulatory BP monitoring (ABPM) every 30 min was an independent predictor of cardiovascular mortality in the general population. Furthermore, Mena et al. [9] reported that high daily BPV assessed by average real variability index of ABPM was associated with the presence and progression of subclinical organ damage, as well as the incidence of cardiovascular events. Therefore, in addition to absolute BP, BPV contributes to better BP management and risk stratification.

Most clinical evidence of BPV has been provided by 24-h ABPM, indicating the importance of daily BPV as a clinical risk index. Various factors including mental stress, behavior, environmental temperature and food/drink intake may contribute to daily BPV [10]. At the same time, the baroreflex is a robust negative feedback system that stabilizes daily BPV through sympathetic modulation [11].

The usefulness of frequency analysis of biological data for the stratification of cardiovascular patients is well known. Heart rate (HR) variability (HRV) is most commonly analyzed in clinical settings. The high-frequency (HF) component of HRV (0.15‒0.4 Hz) reflects the vagal nerve modulated HR change by respiratory fluctuation [12]. On the other hand, the low-frequency (LF) component of HRV is reported to indicate concomitant activity of sympathetic and vagal nerve. In addition, the LF/HF ratio reflects sympatho-vagal balance or the activation of sympathetic nervous system [12]. Frequency analysis focusing on continuous BP recording has been developed using the data of standard catheter-manometer systems or noninvasive plethysmographic devices. The LF component (0.04‒0.15 Hz) of systolic BP (SBP) is also known to be an index of sympathetic modulation directed to blood vessels [13]. Thus, the LF of SBP has been reported to increase during tilt [14, 15], mental stress [15], and even several cardiovascular diseases [16, 17].

The baroreflex is a dominant regulatory system of BPV from seconds to hours [18]. Studies in various animal species have also demonstrated that the baroreflex has a higher gain in the low-frequency range (low-pass characteristics) with the cutoff frequency at around 0.05 Hz [19, 20]. Thus, baroreflex dysfunction increases BPV in the low-frequency range. Our previous study has shown that the slope of power spectral density (PSD) of BP in the frequency range of 0.01 to 0.1 Hz increases with the decrease in baroreflex gain in rats [21]. Therefore, the PSD analysis of BP in the baroreflex frequency range would reflect the baroreflex modulated BPV.

Our goal is to derive new indices to evaluate BPV from very short-term continuous BP recording. As the first step, we analyzed the PSD of very short-term continuous BP recordings from healthy adults in various age groups. Since aging is known to worsen the baroreflex function [10, 22], we hypothesized that aging steepens the slope of PSD of BP in the baroreflex operating frequency range.

## Materials and methods

### Subjects and devices

Between December 2017 and April 2018, we recruited 80 healthy volunteers of various age groups (41 women and 39 men; mean age 57.4, range 28–85 years), who were not prescribed antihypertensive drugs or cardiovascular drugs. We excluded the volunteers who were prescribed drugs for dyslipidemia and diabetes mellitus, because these diseases may affect the circulatory regulation via autonomic or baroreflex dysfunction. None of the subjects presented clinical signs of neurological and autonomic dysfunction or received prescriptions for neurological diseases. Omron Expert Link Co., Ltd. (Kyoto, Japan) was entrusted with recruitment of volunteers. The institutional review board of Omron Healthcare Co., Ltd. (Kyoto, Japan) approved this study, and all participants provided written informed consent to participate.

We used an in-house developed, institutionally approved wearable wrist-type tonometric BP monitor for noninvasive continuous BP recording [10, 23]. We simultaneously recorded electrocardiogram (ECG) and respiratory signal (RESP) from thoracic impedance (BP-A308; Omron Healthcare Co., Ltd., Kyoto, Japan). We also measured oscillometric BP in the upper arm (BP-203RPEIII; Omron Healthcare Co., Ltd., Kyoto, Japan).

### Protocol

**Fig 1:**
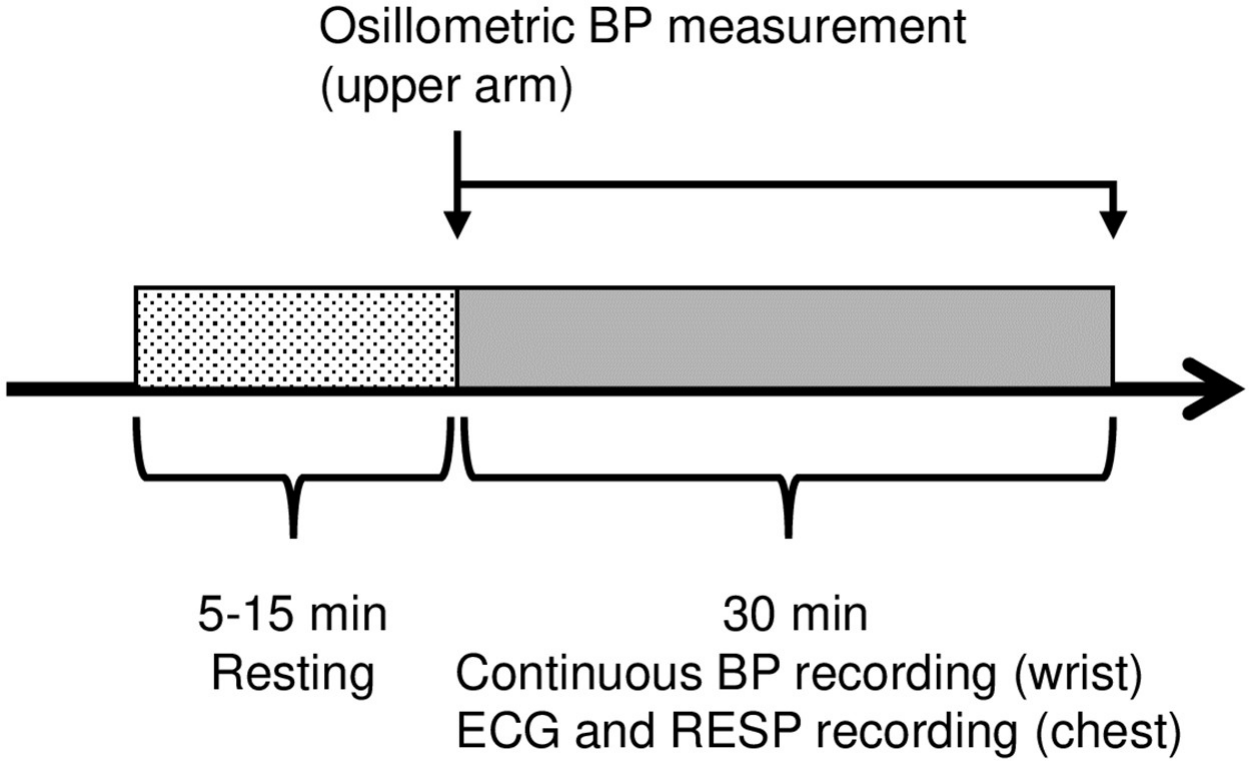
Protocol of this study. After waiting for 5–15 min until all signals were stabilized, continuous BP, ECG and RESP were recorded for 30 min with the subject resting in a supine position. The oscillometric BP was measured before and after the continuous BP recording. BP, blood pressure; ECG, electrocardiogram; RESP, respiratory signal.

We attached the wearable tonometric BP monitor to the wrist and the oscillometric BP monitor to the ipsilateral upper arm. We placed three electrodes for ECG and RESP on the chest. After the preparation, we waited for 5–15 min until all signals were stabilized. We then recorded continuous BP, ECG and RESP for 30 min with the subject resting in a supine position. We measured the oscillometric BP before and after the continuous BP recordings (Fig 1).

### Data analysis

We recruited 80 subjects. Subjects were excluded from analysis if they met any of the following exclusion criteria:

- Subject who showed SBP ≥ 140 mmHg or diastolic BP (DBP) ≥ 90 mmHg (averaged value of oscillometric BP measurements during the protocol).
- Subject who could not complete the stable continuous BP recording for 30 min because of motion, respiration noise and recording failure.
- Subject who had irregular beats more than 10 times during the 30-min recording.

Finally, we excluded 24 subjects from analysis, and studied the remaining 56 subjects.

#### Continuous BP analysis

We digitized continuous BP at 1.0 kHz using a 16-bit analog-to-digital converter (Power Lab 8/35; AD Instruments, Sydney, Australia). We derived SBP (maximum BP), mean BP, DBP (minimum BP) and HR in every beat. We estimated BPV and HRV in the time domain as the SD of beat-by-beat BP (SBP, mean BP and DBP) and HR, respectively, for 30 min.

For frequency analysis of continuous BP, we resampled the mean BP time series at 5 Hz and divided them into 200-second segments with 50% overlap. In each segment, after removing a linear trend, we applied the Hanning window. We applied the fast Fourier transform (FFT) using the Welch’s periodogram [24] and estimated PSD in the frequency range of 0.01 to 0.1 Hz. The integrated PSD area reflects BPV in the frequency range.

We chose this frequency range because previous studies in various animal species indicate that the baroreflex function approximates distinctive low-pass filter characteristics [19, 20]. The baroreflex cannot operate above 0.1 Hz and fully operates at 0.01 Hz; hence the baroreflex strongly attenuates BPV at around 0.01 Hz. To quantify the impact of baroreflex on PSD, we characterized PSD at 0.01 Hz (PSD_0.01Hz_) and 0.1 Hz (PSD_0.1Hz_) and derived the slope of PSD (S1 Fig).

We also assessed baroreflex sensitivity (BRS), which has been extensively used for assessing baroreflex function in clinical settings [25, 26]. BRS is HR response to BP change in the closed-loop condition. In this study, BRS was assessed using the sequence method [27]. BRS was calculated from the slope of linear regression plots of SBP versus RR interval on spontaneous sequences, in which SBP and RR interval concurrently increase (up sequences) or decrease (down sequences) for three or more consecutive beats.

#### Statistical analysis

We performed statistical analyses using commercially available software (BellCurve for Excel version 3.21, Social Survey Research Information Co., Ltd., Tokyo, Japan). We used Pearson correlation coefficients to assess the relationship between age and each measurement variable for all age groups. We divided the subjects into four age groups: 26–40, 41–55, 56–70 and 71–85 years. We compared all age groups by one-way factorial analysis of variance (ANOVA) followed by the Tukey-Kramer test. We considered differences to be statistically significant at p < 0.05.

## Results

### Baseline characteristics

Table 1 shows the baseline characteristics. The proportion of males was relatively low in the 26–40 age group. Body mass index (BMI) did not differ among age groups. In this cohort, SBP and DBP increased slightly with age (S2 Fig).

**Table 1:** Baseline characteristics and BP stratified by age group.

	Total	Age groups (years)				p value
		26–40	41–55	56–70	71–85	
No. of subject	56	14	15	17	10	-
Age (years)	54.3±16.9	32.9±3.93	47.7±3.96	62.6±4.70	80.0±3.71	-
Male (%)	48.2	35.7	46.7	58.8	50.0	NS
BMI (kg/m^2^)	21.1±3.23	20.0±3.40	20.6±3.31	22.0±3.24	22.1±2.54	NS
SBP (mmHg)	111.7±12.6	102.1±8.61	110.8±10.2	113.1±13.3	124.4±8.22	p < 0.01
DBP (mmHg)	66.6±9.24	59.9±4.21	67.5±9.41	68.0±10.4	72.2±7.46	p < 0.01

SBP and DBP were measured by an oscillometric BP monitor. The SBP and DBP values were obtained by averaging oscillometric BP before and after the 30-min continuous BP recording. Values are expressed as mean ± SD. One-way factorial ANOVA (BMI, SBP and DBP) and Pearson’s χ^2^ test (percent males) were used for comparison among four groups.

BP, blood pressure; BMI, body mass index; SBP, systolic blood pressure; DBP, diastolic blood pressure; NS, not significant; SD, standard deviation; ANOVA, analysis of variance.

### Impact of aging on BP and BPV from continuous BP recordings

**Fig 2:**
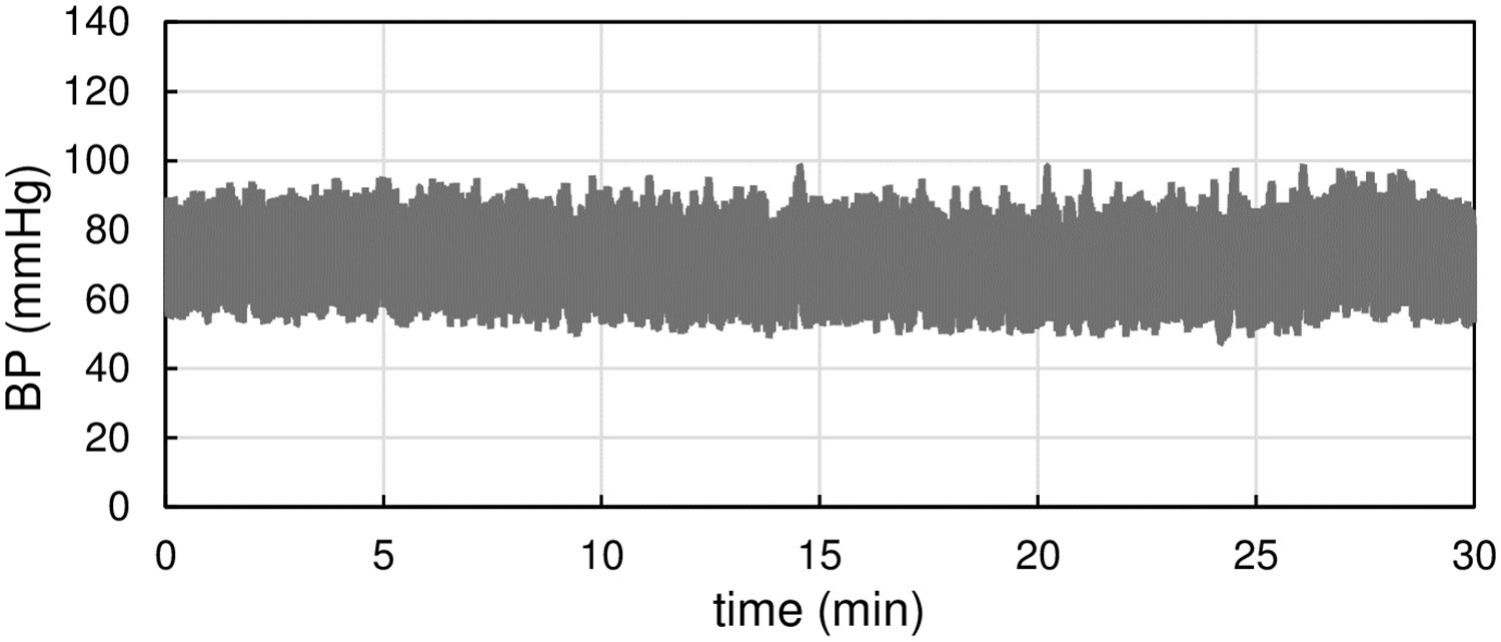
Representative Blood Pressure (BP) time series of 30-min continuous tonometric BP recording.

Shown in Fig 2 is a representative time series of continuous tonometric BP recording for 30 min. Both SBP and DBP fluctuated continuously without a large change in mean BP. Averaged BP and HR did not differ among the four age groups (Table 2). SBP increased slightly with age (S3 Fig).

**Table 2:** Averaged BP and HR obtained from 30-min continuous recordings.

	Total	Age groups (years)				P value
		26–40	41–55	56–70	71–85	
No. of subject	56	14	15	17	10	
SBP (mmHg)	107.1±14.7	100.8±11.8	106.7±12.9	109.4±17.3	112.5±14.9	NS
mean BP (mmHg)	78.4±11.7	74.8±7.88	78.4±12.1	81.1±14.6	78.8±10.6	NS
DBP (mmHg)	61.0±10.9	60.4±6.87	61.3±12.1	62.9±13.1	57.9±10.7	NS
HR (bpm)	63.4±10.6	62.5±9.94	64.2±13.5	60.5±8.65	68.7±8.63	NS

Continuous BP was recorded by a wearable tonometric BP monitor and HR by a 3-electrode biological monitor for 30 min. After deriving SBP (maximum BP), mean BP, DBP (minimum BP) and HR in every beat, means of beat-by-beat BP (SBP, mean BP and DBP) and HR for 30 min were estimated. Values are expressed as mean ± SD. One-way factorial ANOVA was used for comparison among four groups.

BP, blood pressure; HR, heart rate; SBP, systolic blood pressure; DBP, diastolic blood pressure; NS, not significant; SD, standard deviation; ANOVA, analysis of variance.

**Fig 3:**
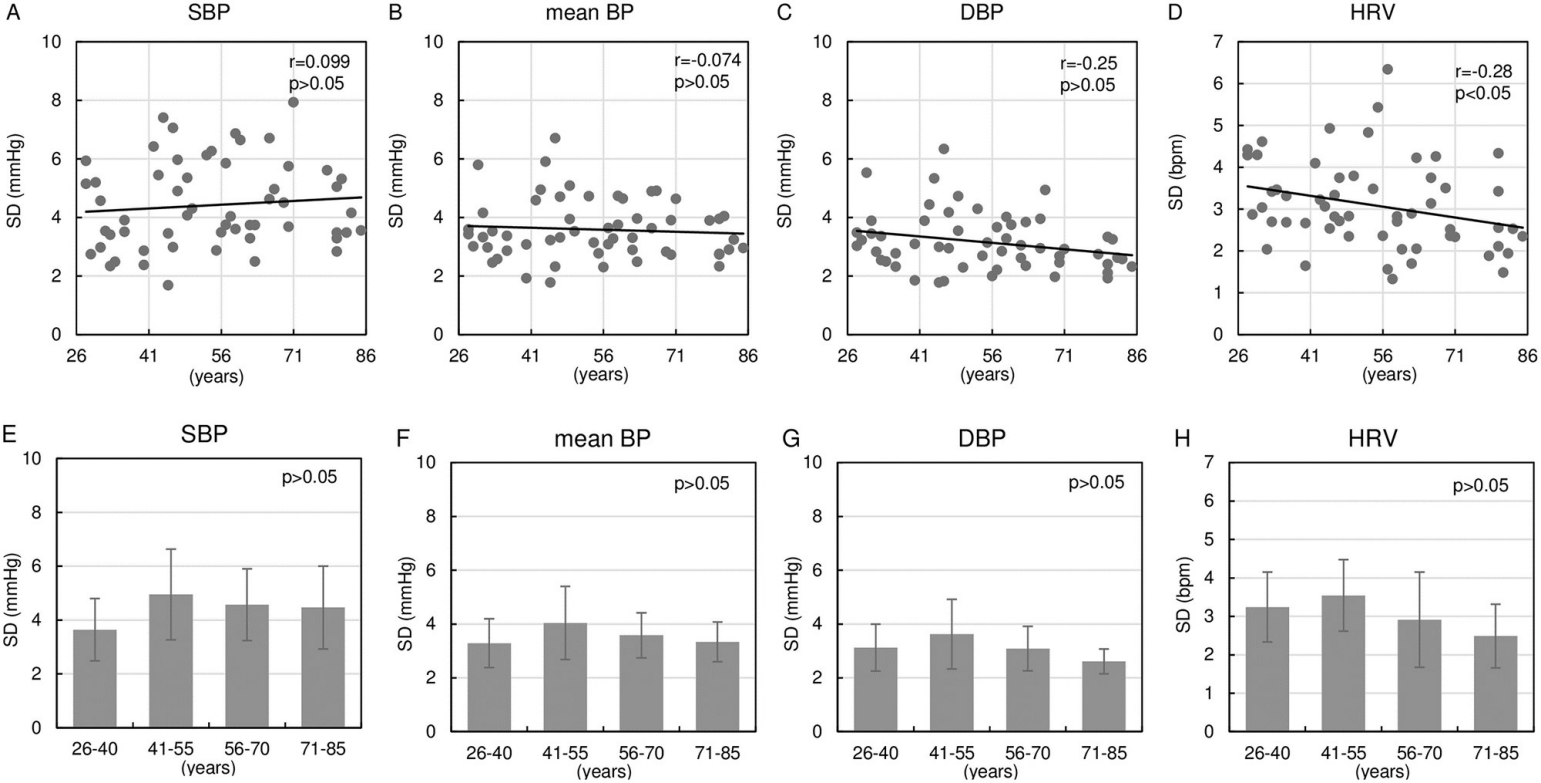
Impact of aging on BPV and HRV. The relationship of age with BPVs and HRV: individual data (A-D) and group data (E-H). SD of beat-by-beat SBP, mean BP, DBP and HR were calculated. Upper panels present scatter plots of individual data for the relationship of age with BPVs and HRV analyzed by Pearson’s correlation coefficient (r). Straight lines indicate linear regression lines. Lower panels present the comparisons of BPVs and HRV among four age groups analyzed by one-way factorial ANOVA. BPV, blood pressure variability; HRV, heart rate variability; SBP, systolic blood pressure; BP, blood pressure; DBP, diastolic blood pressure; HR, heart rate; SD, standard deviations; ANOVA, analysis of variance.

Fig 3 demonstrates the relationship of age with BPVs and HRV expressed by SD. The upper panels show the scatter plots of individual data between age and BPVs or HRV. While BPVs did not correlate significantly with age, HRV correlated weakly with age and tended to decrease with aging. The lower panels compare the BPVs and HRV among four age groups. There were no significant differences in BPVs and HRV among the four groups.

**Fig 4:**
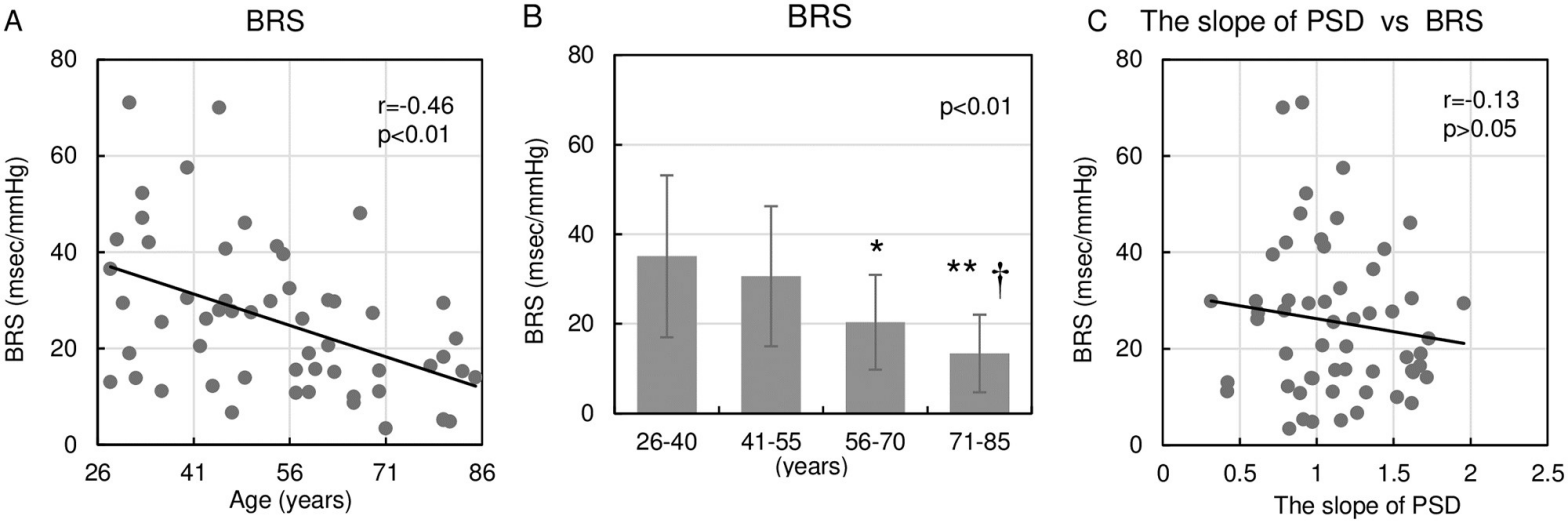
Relationship between age, BRS and the slope of PSD. (A): Scatter plot of individual data for the relationship between age and BRS. Straight line indicates linear regression line. Pearson correlation coefficient (r) was used to assess the goodness-of-fit of the linear regression. (B): Age group comparison of BRS. One-way factorial ANOVA followed by Tukey-Kramer test was used to compare the relationship among four age groups. Data are shown as mean ± SD. (C): Scatter plot of individual data for the relationship between the slope of PSD and BRS. BRS did not correlate significantly with the slope of PSD. *p < 0.05 versus 26–40 age group. **p < 0.01 versus 26–40 age group. †p < 0.05 versus 41–55 age group. BRS, baroreflex sensitivity; PSD, power spectrum density; ANOVA, analysis of variance; SD, standard deviations.

Fig 4 demonstrates the relationship between age and BRS, and the relationship between the slope of PSD and BRS. Fig 4A shows the scatter plot of individual data between age and BRS. Fig 4B compares BRS among the four age groups. BRS correlated significantly with age and tended to decrease as age increases (Fig 4A). BRS was significantly smaller in the 71–85 age group than in the 26–40 and 41–55 age groups (Fig 4B). On the other hand, BRS did not correlate significantly with the slope of PSD (Fig 4C).

### Impact of aging on characteristics of PSD of BP

**Fig 5:**
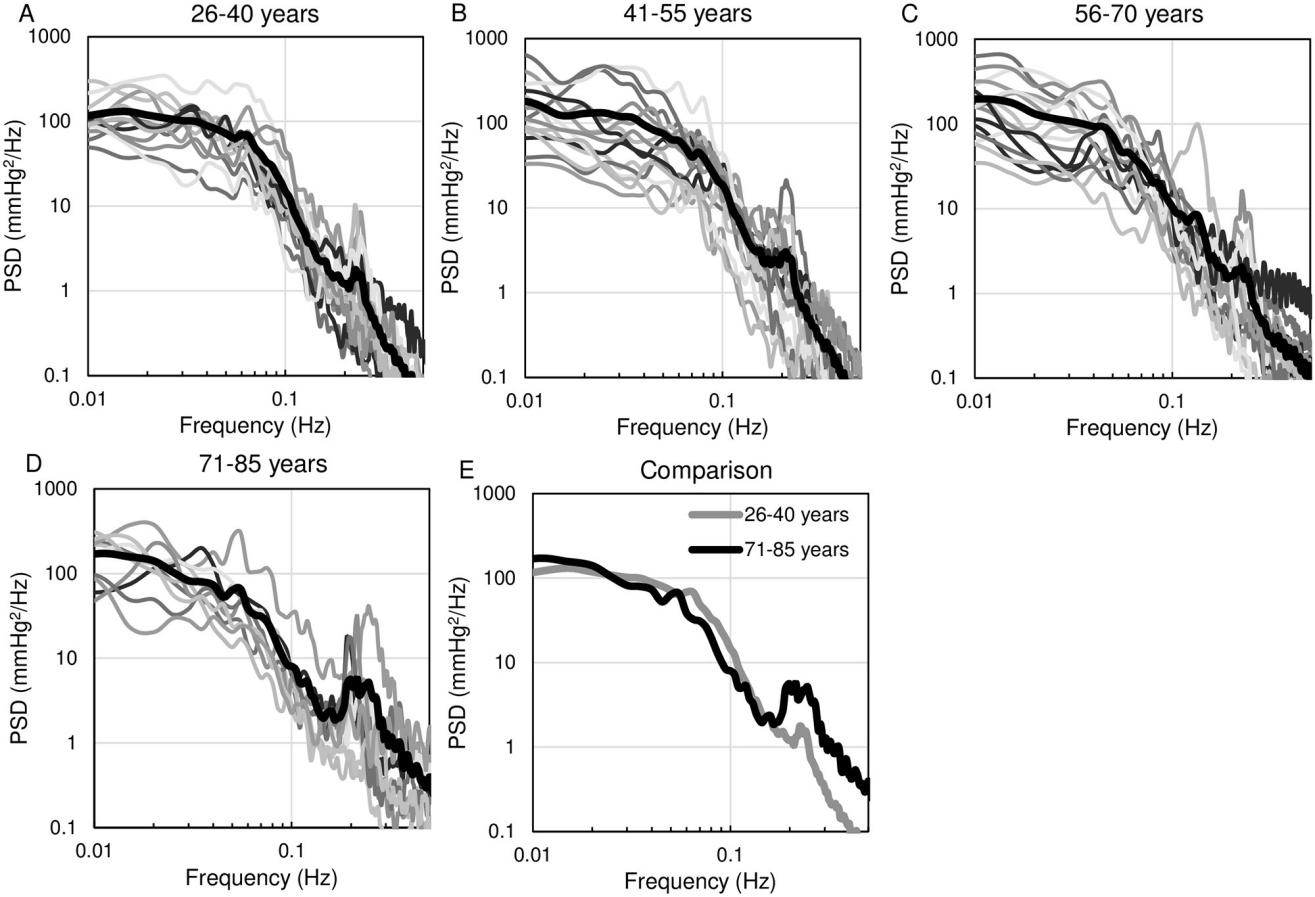
The PSD estimated from 30-min tonometric continuous BP recordings in each age group. (A-D): Individual (fine lines) and mean PSD (bold line) estimated from 30-min continuous BP recordings. (E): Comparison of mean PSD between the 26–40 and 71–85 age groups. The slope of PSD between 0.01 and 0.1 Hz was steeper in the 71–85 age group than in the 26–40 age group. PSD, power spectrum density; BP, blood pressure.

Fig 5 shows the individual and group PSD estimated from 30-min continuous BP recordings. Both PSD and frequency axes are logarithmically scaled. In all age groups, PSD was relatively flat around 0.01 Hz and decreased as the frequency increased. As shown in Fig 5E, the slope of PSD between 0.01 and 0.1 Hz in the 71–85 age group was steeper than that in the 26–40 age group.

**Fig 6:**
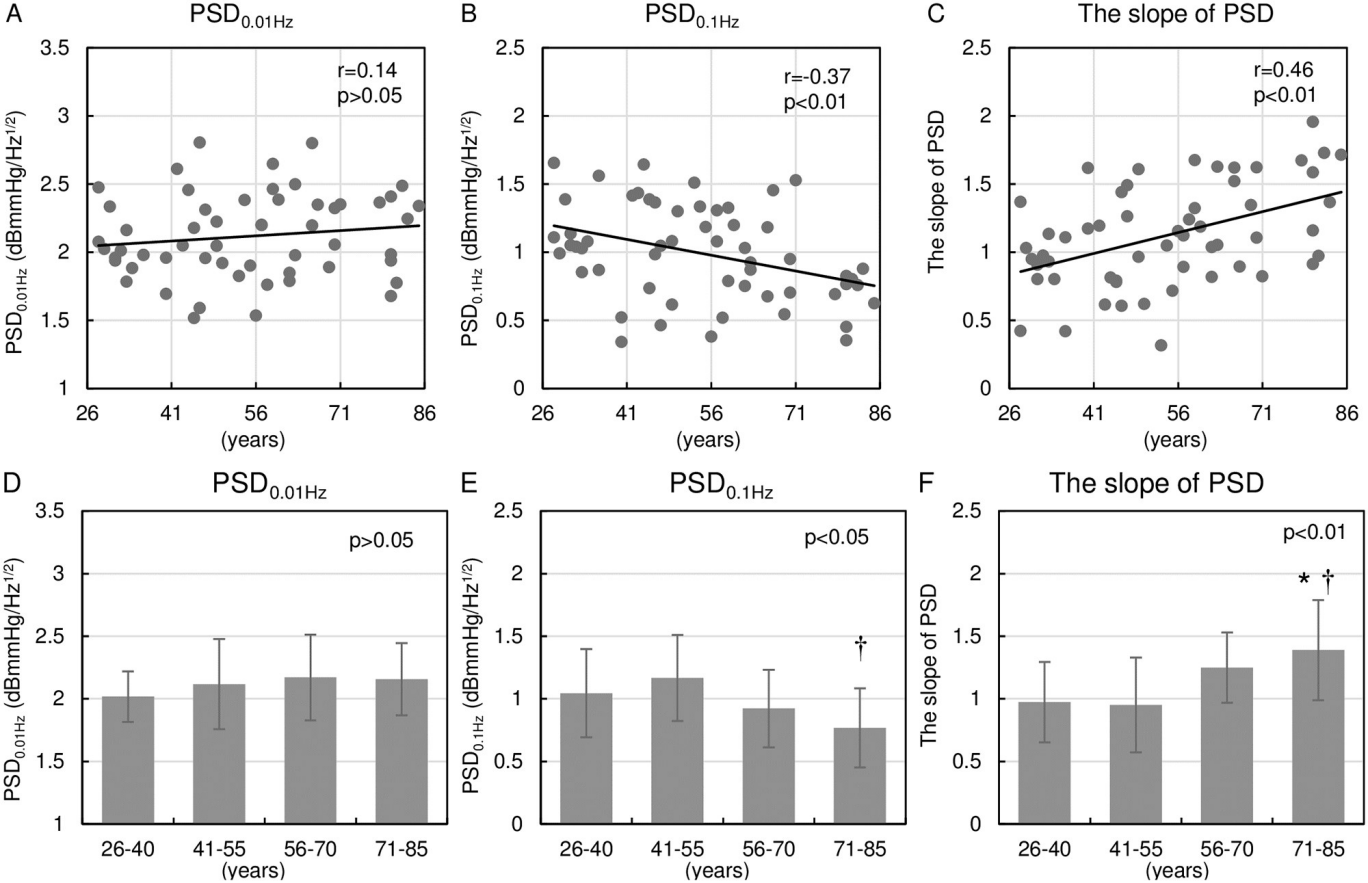
Relationship of age with PSD characteristics. (A-C): Scatter plots of individual data for the relationship between age and PSD characteristics. Straight lines indicate linear regression lines. Pearson correlation coefficient (r) was used to assess the goodness of fit of the linear regression. (D-F): Age group comparison of PSD characteristics. One-way factorial ANOVA followed by the Tukey-Kramer test was used to compare the relationship among four age groups. Data are shown as mean ± SD. The slope of PSD between 0.01 to 0.1 Hz correlated significantly with age. *p < 0.05 versus 26–40 age group. †p < 0.05 versus 41–55 age group. PSD, power spectrum density; ANOVA, analysis of variance; SD, standard deviations.

Fig 6 demonstrates the relationship of age with PSD characteristics. The upper panels show scatter plots of individual data between age and PSD characteristics. The lower panels compare PSD characteristics among the four age groups. PSD_0.01Hz_ did not correlate with age in individual data (Fig 6A) or the group data (Fig 6D). On the other hand, PSD_0.1Hz_ correlated significantly with age and tended to decrease as age increased (Fig 6B). As a result, the slope of PSD between 0.01 and 0.1 Hz correlated significantly with age and tended to increase as age increased (Fig 6C). The slope of PSD between 0.01 and 0.1 Hz was significantly larger in the 71–85 age group than in the 26–40 and 41–55 age groups (Fig 6F).

The PSD characteristics between males and females in each age group was also compared. Although the sample size was small, the slope of PSD within the same age group did not differ significantly between males and females. The general trend of age-related alteration in the slope of PSD was also the same between males and females (S1 Table, S4 and S5 Figs).

## Discussion

This study aimed to investigate in healthy human subjects the very short-term BPV focusing on the baroreflex operating frequency range between 0.01 and 0.1 Hz. Major findings of this study include: (1) we can stably estimate PSD of BP from 30-min continuous BP recording in the baroreflex operating frequency range; (2) PSD of BP showed low-pass filter characteristics; (3) aging significantly steepened the slope of PSD of BP; (4) aging did not alter SD of BP time series, which indicate BPV in the time domain.

### Characteristics of PSD estimated from 30-min continuous BP recording in humans

The PSD of BP in healthy human subjects was nearly flat around 0.01 Hz and decreased gradually with the increase in frequency. We previously reported that the PSD in rats decreased with the increase in frequency with an inflection point at around 0.1 Hz [21]. In both humans and rats, PSD of BP decreased in the frequency range between 0.01 to 0.1 Hz. However, the precise shape of the PSD differs between the two species, especially the flatness around 0.01 Hz in humans and the inflection point at around 0.1 Hz in rats. Since the baroreflex dynamic function, which is the major regulator of BPV in the frequency range of 0.01 to 0.1 Hz, is similar in various animal species [19, 20, 28], we speculate that factors such as the effect of respiration on BP and neural disturbance may contribute to the differences in the shape of the PSD of BP. Further investigation is needed to characterize the PSD of BP in humans.

The PSD analysis of continuous BP recording provides insight into how BPV relates to autonomic regulation. Castiglioni et al. [29] reported that PSD between 0.0001 and 0.1 Hz exhibited low-pass filter characteristics in human subjects. Our observation is consistent with the previous report that showed the frequency-dependent attenuation of PSD in humans. To characterize baroreflex-regulated BPV, we focused on the PSD in the frequency range above 0.01 Hz.

### Impact of aging on characteristics of PSD of BP

We previously reported that a decrease in baroreflex total loop gain increases the slope of PSD of BP in the frequency range between 0.01 and 0.1 Hz in rats. Thus, we have shown that the PSD slope is reciprocal to the baroreflex open-loop gain [21]. Conci et al. [30] reported that brain death steepened the slope of PSD of BP, indicating compromised autonomic regulation of BPV. Omboni et al. [31] reported that the slope of PSD between 0.01 to 0.1 Hz was higher in patients with autonomic dysfunction than in healthy subjects. These findings indicate that the slope of PSD of BP in the frequency range of 0.01 to 0.1 Hz closely reflects the underlying baroreflex function.

To evaluate the clinical utility of PSD analysis of 30-min continuous BP recording, we examined the relationship of PSD with aging. It is well known that aging impairs baroreflex function, one of the most powerful mechanisms in regulating BPV. Omboni al. [31] also reported that PSD of BP in older subjects showed lower power at around 0.1 Hz and higher power between 0.02 and 0.07 Hz, compared to younger subjects. Orthostatic hypotension and postprandial hypotension are common pathophysiology in older people [32–34], and aging-related autonomic dysfunction could play a significant role in worsening hypotension. Aging-related atherosclerosis and reduced arterial distensibility may blunt the baroreflex afferent loop function and result in baroreflex dysfunction [35]. Interestingly, only the frequency domain analysis of BP recording can detect the alteration of BPV with aging.

In this study, we found no significant correlation between age and SD of BP, indicating that aging does not significantly alter BPV in healthy subjects. PSD_0.01Hz_ did not change significantly with age, while PSD_0.1Hz_ was significantly reduced in older subjects. We previously reported that left ventricular (LV) dysfunction induced by myocardial infarction decreased integrated PSD above 0.01 Hz [21]. Since PSD is the Fourier transform of the variance of BP time series, integrated PSD is proportional to BP variance in this frequency range. Berry et al. [36] also reported that ABPM-assessed BPV decreased in heart failure patients. Thus, we need to interpret the amount of BPV carefully considering cardiac function, especially when evaluating patients with depressed cardiac function.

BRS is a useful method for the estimation of baroreflex function. Tank et al. [37] and Boettger et al. [38] reported that BRS was significantly lower in older than in younger subjects. As shown in Fig 4, BRS clearly decreased in an age-dependent manner, while there was no correlation between BRS and the slope of PSD. Since BRS is a HR response to BP change and reflects the cardiac vagal baroreflex, the slope of PSD, which strongly enhances the baroreflex pressure stabilizing function, is not equal to BRS. Further investigations may be needed to clarify the difference in the major determinant of age-related changes in BRS and the slope of PSD.

The renin-angiotensin-aldosterone system (RAAS) may also regulate BPV strongly. In older people, the secretion of aldosterone increases, while the renin activity decreases. This results in a blunted ability to secrete aldosterone against changes in renal blood flow, such as in sodium restriction [39]. Hence, RAAS dysregulation in older people worsens hormonal BP regulation and increases BPV. In addition, inadequate RAAS activation also increases BP and deteriorates baroreflex function through activation of sympathetic nerve activity [40]. Therefore, age-related RAAS dysregulation may steepen the slope of PSD in older subjects. Further investigations may be needed to deepen the understanding of age-related alteration of very short-term BPV in terms of RAAS regulation.

### Clinical application of very short-term BPV analysis

In this study, we recorded continuous BP for 30 min and estimated the PSD in the baroreflex operating frequency range. The PSD analysis enables us not only to estimate BPV from the very short-term data but also characterize the BPV regulatory system, the baroreflex system. As mentioned above, BPV is conventionally evaluated by oscillometric devices. Since 24-h ABPM and visit-to-visit BP study assess the variability of intermittent BP measurements, these methods cannot address the frequency characteristics of BPV in the baroreflex operating frequency range. In addition, these methods require long-term device attachment (at least during the night) or a prolonged period of observation (at least three visits), which pose a burden on patients. Our proposed method has the potential as a novel measurement of BPV because it is noninvasive, and the recording time is only 30 min. The PSD analysis makes it possible to stratify BPV considering the BPV regulatory system.

The clinical application of the PSD method requires further investigation. Although the PSD evaluation of BPV requires much shorter time of BP recordings, the 30-min BP recording remains too long as a clinical tool in routine patient care. Thus, we need to develop a novel algorithm to estimate PSD of BP from shorter (such as 5-min) BP recordings without losing the accuracy of PSD estimation.

Although the very short-term BPV analysis has the potential for stratifying the CV risk as shown by several animal studies, the predictive power is not as high as daily BPV assessed by 24-h ABPM. The major reason for the lower predictive power of very short-term BPV may be the lack of clinical evidence [41, 42]. Since the very short-term BPV analysis requires continuous BP measurement devices, the development of those devices will change this situation. In this study, we recorded continuous BP noninvasively using an in-house developed wearable wrist-type BP monitor based on arterial tonometry [10, 23]. Our algorithm of BPV analysis can be applied to any continuous BP recording system. Recently, the field of continuous BP monitor is developing rapidly due to the increasing demand for high-performance healthcare devices and optimal patient management [43]. The volume clamp method (FMS-Finapres Medical System, Arnhem, the Netherlands; CNSystems Medizintechnik AG, Graz, Austria), which can capture instantaneous BP waveform at the finger, has been used for recording continuous BP in clinical settings [44]. As for cuffless continuous noninvasive BP monitors, the Visi Mobile System (Sotera Wireless, CA, USA) based on the pulse transit time method has been shown to provide acceptable BP recording during the long-term attachment [45]. Similar wearable devices such as Caretaker (Caretaker Medical LLC, VA, USA) and BB-613 (Biobeat Technologies LTD, Israel) have been approved as medical devices in the US. With such diverse device development, translation of our method to clinical application would be rather soon.

### Limitations

There are several limitations to this study. First, the number of subjects included in this study was relatively small, thus limiting statistical power. In addition, individual variation of PSD of BP was observed in each age group. This variation makes detailed analysis of PSD difficult. Thus, we need to investigate a larger number of healthy subjects to further understand the PSD of BP.

Second, we conjectured that the slope of PSD of BP reflects aging-induced baroreflex failure. Thus, we need to compare the baroreflex function by direct measurement to the data obtained by our proposed method. In addition, autonomic challenges such as cold pressure test, handgrip stress and vasoactive drug administration may deepen the understanding of autonomic function including baroreflex. However, we focused on very short-term BPV with a clinically applicable protocol without using such intervention. Further investigations are also needed to address this issue.

Lastly, the ultimate goal of this study is to derive indices from the PSD of BP for clinical risk stratification. As the first step to achieve this goal, we excluded patients with cardiovascular diseases in this study. In the next step, we need to clarify the significance of PSD-derived indices in patients with cardiovascular diseases, especially hypertension.

## Conclusions

The PSD analysis of 30-min continuous BP recording characterizes the very short-term BPV in healthy human subjects. Aging steepens the slope of PSD of BP without changing the magnitude of BPV. Aging-related baroreflex impairment may contribute to the increase in the slope of PSD of BP.

## Supporting information

S1 Fig The PSD characteristics evaluated in this study. (PDF)

S2 Fig Impact of aging on baseline characteristics and BP. (PDF)

S3 Fig Impact of aging on averaged BP and HR from 30-min continuous recordings. (PDF)

S4 Fig Impact of gender on baseline characteristics and BP. (PDF)

S5 Fig Impact of gender on characteristics of PSD of BP. (PDF)

S1 Table Impact of gender on baseline characteristics and BP. (PDF)

## Abbreviations

ABPM: ambulatory blood pressure monitoring
ANOVA: analysis of variance
BMI: body mass index
BP: blood pressure
BPV: blood pressure variability
BRS: baroreflex sensitivity
DBP: diastolic blood pressure
ECG: electrocardiogram
FFT: Fast Fourier Transform
HF: high-frequency
HR: heart rate
HRV: heart rate variability
LF: low-frequency
LV: left ventricular
NS: not significant
PSD: power spectrum density
RAAS: renin-angiotensin-aldosterone system
RESP: respiratory signal
SBP: systolic blood pressure
SD: standard deviation
