# Supplementary material for: Aging steepens the slope of power spectrum density of 30-minute continuous blood pressure recording in healthy human subjects
Source: PLoS One. 2021 Mar 18;16(3):e0248428. doi: 10.1371/journal.pone.0248428 (PMC7971546; doi:10.1371/journal.pone.0248428)
Supplement: S1 Fig — (PDF) [file pone.0248428.s001.pdf]

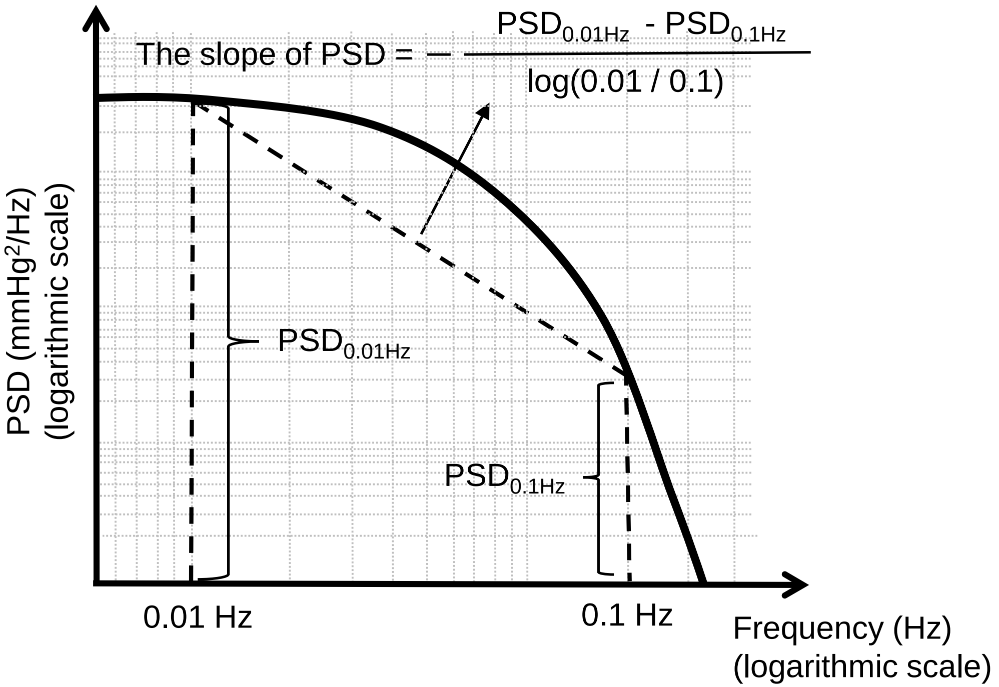

The PSD characteristics evaluated in this study.

To characterize PSD, we focused on the three parameters. $PSD_{0.01Hz}$ is the log base 10 of PSD at 0.01 Hz, and $PSD_{0.1Hz}$ is that at 0.1 Hz. We also calculated the slope of PSD between 0.01 Hz and 0.1 Hz on a double logarithmic scale.

PSD, power spectrum density.
