# Supplementary material for: Aging steepens the slope of power spectrum density of 30-minute continuous blood pressure recording in healthy human subjects
Source: PLoS One. 2021 Mar 18;16(3):e0248428. doi: 10.1371/journal.pone.0248428 (PMC7971546; doi:10.1371/journal.pone.0248428)
Supplement: S3 Fig — (PDF) [file pone.0248428.s003.pdf]

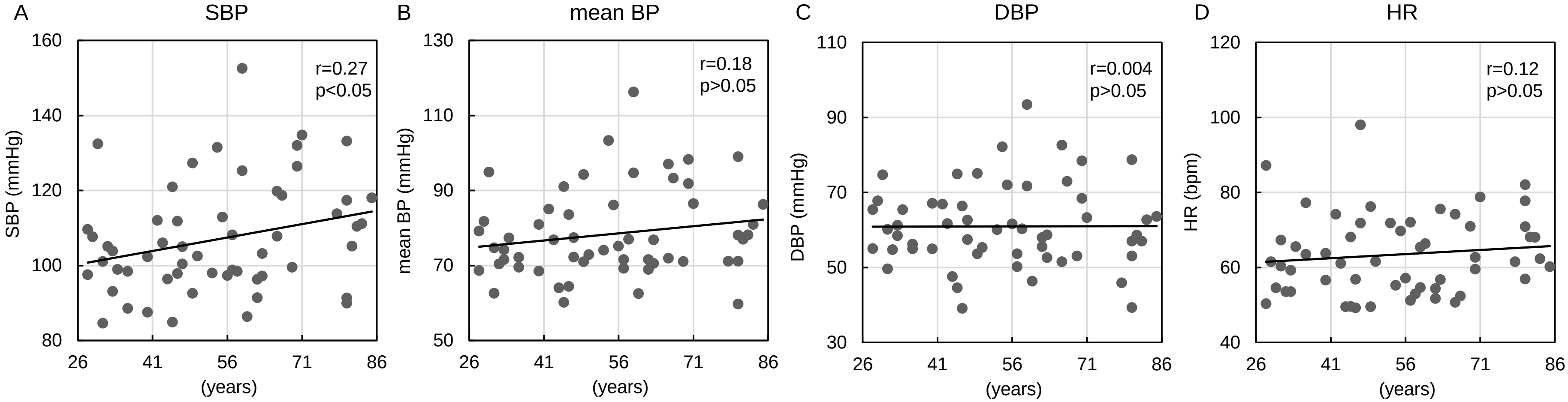

Impact of aging on averaged BP and HR obtained from 30-min continuous recordings.

Scatter plots of individual data for the relationship of age versus SBP, mean BP, DBP and HR analyzed by Pearson's correlation coefficient (r). Straight lines indicate linear regression lines. Continuous BP was recorded by a wearable tonometric BP monitor and HR by a 3-electrode biological monitor for 30 min. After deriving SBP (maximum BP), mean BP, DBP (minimum BP) and HR in every beat, means of beat-by-beat BP (SBP, mean BP, and DBP) and HR were estimated for 30 min. BP, blood pressure; HR, heart rate; SBP, systolic blood pressure; DBP, diastolic blood pressure.
