# Supplementary material for: Aging steepens the slope of power spectrum density of 30-minute continuous blood pressure recording in healthy human subjects
Source: PLoS One. 2021 Mar 18;16(3):e0248428. doi: 10.1371/journal.pone.0248428 (PMC7971546; doi:10.1371/journal.pone.0248428)
Supplement: S4 Fig — (PDF) [file pone.0248428.s004.pdf]

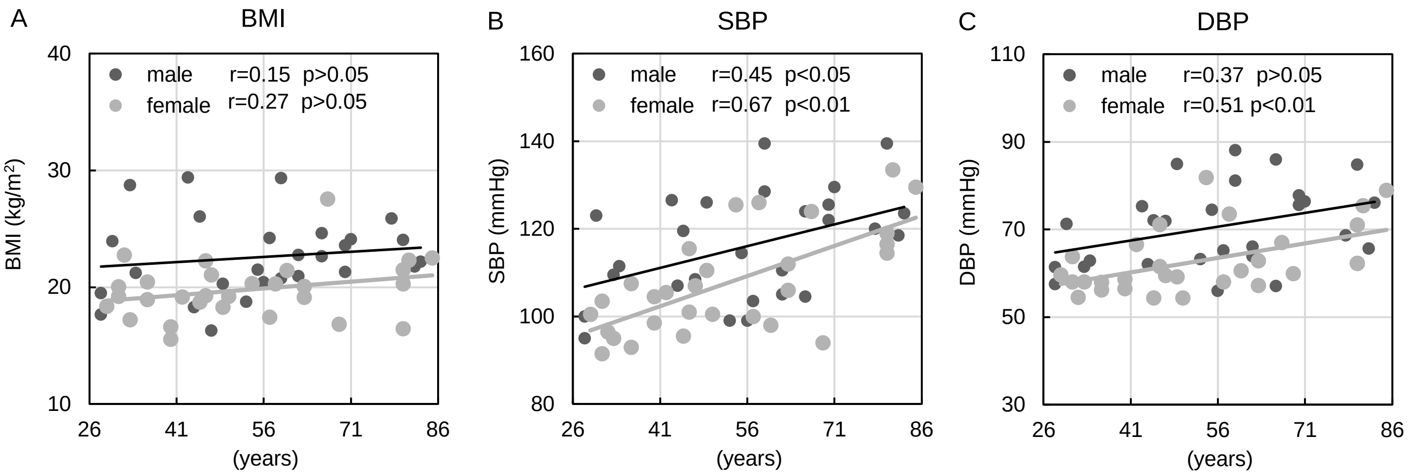

Impact of gender on baseline characteristics and BP.

Scatter plots of individual data for the relationship of age versus BMI, SBP and DBP analyzed by Pearson's correlation coefficient (r) for males and females. Straight lines indicate linear regression lines. SBP and DBP were measured by an oscillometric BP monitor. The SBP and DBP values were obtained by averaging oscillometric BP before and after the 30-min continuous BP recording.

BP, blood pressure; BMI, body mass index; SBP, systolic blood pressure; DBP, diastolic blood pressure.
