# Supplementary material for: Aging steepens the slope of power spectrum density of 30-minute continuous blood pressure recording in healthy human subjects
Source: PLoS One. 2021 Mar 18;16(3):e0248428. doi: 10.1371/journal.pone.0248428 (PMC7971546; doi:10.1371/journal.pone.0248428)
Supplement: S5 Fig — (PDF) [file pone.0248428.s005.pdf]

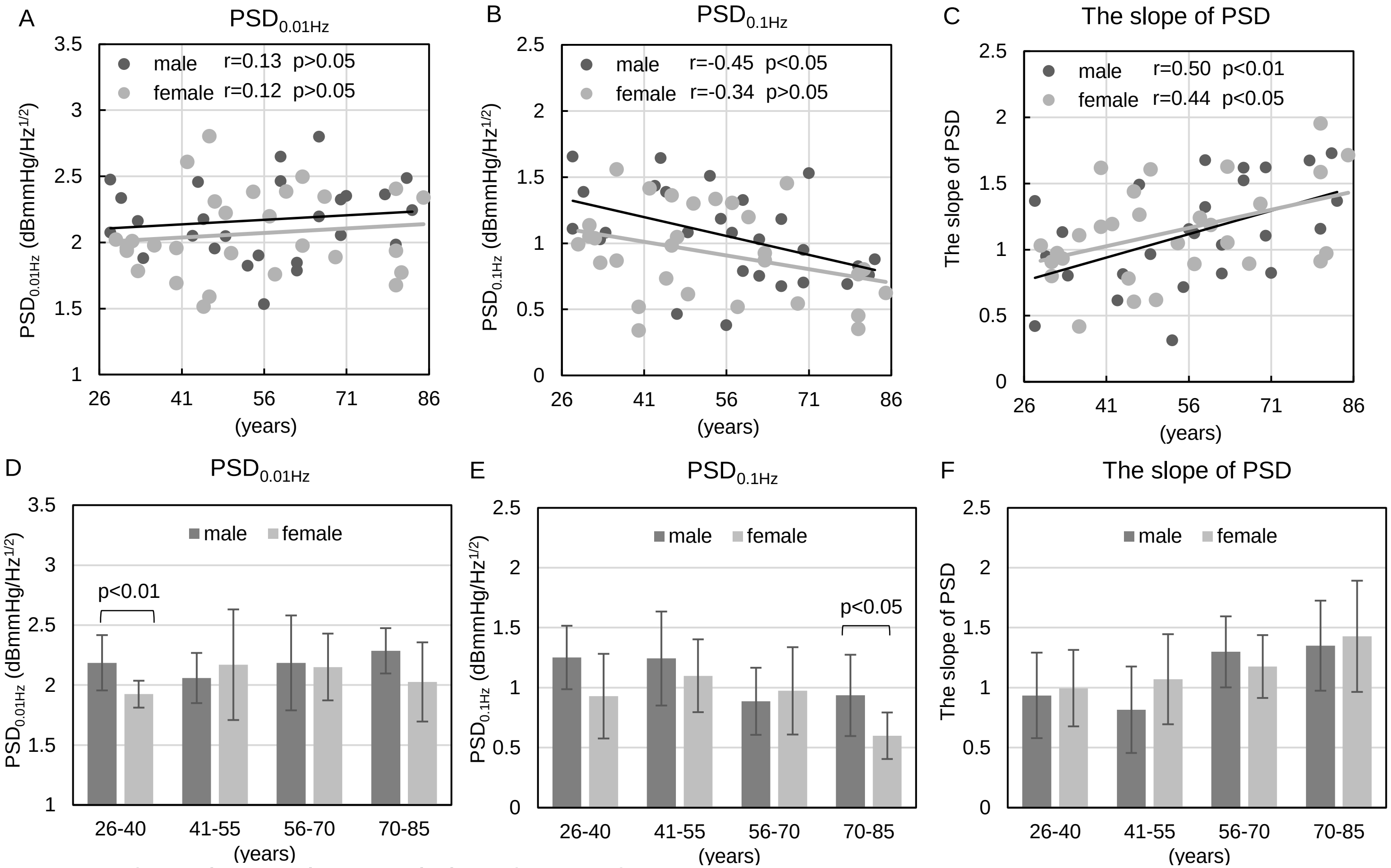

Impact of gender on characteristics of PSD of BP.

(A-C): Scatter plots of individual data for the relationship between age and PSD characteristics for males and females. Straight lines indicate linear regression lines. Pearson correlation coefficient (r) was used to assess the goodness-of-fit of the linear regression. (D-F): Age group comparison of PSD characteristics for males and females. Student's t-test was used to compare males and females in each age group. Data are shown as mean ± SD. $PSD_{0.01Hz}$ in males of 26-40 age group and $PSD_{0.1Hz}$ in males of 71-85 age group were significantly higher than those in females of the corresponding age groups (D and E). The trend in the slope of PSD did not differ between males and females (F).

PSD, power spectrum density; SD, standard deviations.
