# Supplementary material for: Aging steepens the slope of power spectrum density of 30-minute continuous blood pressure recording in healthy human subjects
Source: PLoS One. 2021 Mar 18;16(3):e0248428. doi: 10.1371/journal.pone.0248428 (PMC7971546; doi:10.1371/journal.pone.0248428)
Supplement: S1 Table — (PDF) [file pone.0248428.s006.pdf]

	Total	Age groups (years)											
		26-40			41-55			56-70			71-85		
		M	F	p	M	F	p	M	F	p	M	F	p
No. of subject	56	5	9	-	7	8	-	10	7	-	5	5	-
Age (years)	54.3 ±16.9	30.6 ±2.79	34.2 ±3.99	$p < 0.05$	48.0 ±4.58	47.4 ±3.62	NS	62.7 ±5.10	62.4 ±4.47	NS	78.8 ±4.76	81.2 ±2.17	NS
BMI (kg/m^2)	21.1 ±3.23	22.2 ±4.32	18.8 ±2.19	$p < 0.05$	21.5 ±4.65	19.8 ±1.33	NS	23.1 ±2.65	20.4 ±3.54	$p < 0.05$	23.6 ±1.66	20.6 ±2.49	$p < 0.05$
SBP (mmHg)	111.7 ±12.6	107.8 ±10.9	98.9 ±5.47	$p < 0.05$	114.4 ±10.3	107.6 ±9.53	NS	116.2 ±13.5	108.6 ±12.6	NS	126.2 ±8.56	122.6 ±8.40	NS
DBP (mmHg)	66.6 ±9.24	62.9 ±5.10	58.2 ±2.60	$p < 0.05$	72.0 ±7.76	63.6 ±9.35	$p < 0.05$	71.7 ±11.6	62.8 ±5.78	$p < 0.05$	74.3 ±7.51	70.0 ±7.57	NS

Impact of gender on baseline characteristics and BP.

Values are expressed as mean ± SD. Student’s t-test were used to compare between males and females in each age group. BMI, SBP and DBP in males of 26-40 age group were significantly higher than those in females of the corresponding age groups.

M, male; F, female; BP, blood pressure; BMI, body mass index; SBP, systolic blood pressure; DBP, diastolic blood pressure; NS, not significant; SD, standard deviation.
